# 

**Published:** 1889-02

**Authors:** 


CONTENTS.

ORIGINAL COMMUNICATIONS—

Chronic Pelvic Cellulitis and the Conditions which Simulate it. By Virgil O. Hardon, M. D., Atlanta., Ga..................... 715

Two Cases of Medico-Legal Interest. By H. N. Moyer, M. D., Chicago, Ill ........................................................................ 728

What shall the Surgeon do with the Urethra and the Testes in Cases of Amputation of the Penis. By W. Locke Chew., M. D., Birmingham, Ala ............................................ 730

Constipation a Cause of Catarrh. By N. R. Gordon, M. D., Springfield, Ill.......................................................................... 740

Foreign Bodies in the Nose. By Frank Trester Smith, M. D., Chattanooga, Tenn.................................................................. 742

Treatment of Club-foot by Means of the Thomas Wrench, with Report of Cases. By V. P. Gibney, M. D., New York..... 749

SOCIETY REPORTS—

Atlanta Society of Medicine.............................................. 755

Allegheny County Medical Society.................................... 758

CORRESPONDENCE—

Our New York Letter.......................................................... 769

Quarantine Conference...................................................... 771

EDITORIAL—

Retrospect.......................................................................... 774

Items................................................................................... 775

